# Supplementary material for: Diagnostic accuracy of Truenat Tuberculosis and Rifampicin-Resistance assays in Addis Ababa, Ethiopia
Source: PLoS One. 2021 Dec 28;16(12):e0261084. doi: 10.1371/journal.pone.0261084 (PMC8714111; doi:10.1371/journal.pone.0261084)
Supplement: Attachment — Submitted filename: Response to Reviewers.docx [file pone.0261084.s001.docx]

Response to Reviewers with regard to PONE-D-21-22469 revision

Dear PLOS ONE Editorial team

Thank you for the in-depth review and comments, which we agree will improve overall quality of the manuscript. Please find below a point-by-point response to editor for journal requirements and to each of the reviewers’ for comments. Therefore, we submitted a revised version of the manuscript and a revised manuscript with track changes.

Kind regards,

Abyot Meaza, Corresponding author and PI of Truenat Evaluation Study

--------------------------------------------

I. Journal requirements:

When submitting your revision, we need you to address these additional requirements.

1. Please ensure that your manuscript meets PLOS ONE's style requirements, including those for file naming. The PLOS ONE style templates can be found at

<https://journals.plos.org/plosone/s/file?id=wjVg/PLOSOne_formatting_sample_main_body.pdf> and

<https://journals.plos.org/plosone/s/file?id=ba62/PLOSOne_formatting_sample_title_authors_affiliations.pdf>

The manuscript revised to meet PLOS ONE's style requirements, including those for file naming as per the comment. Title, Title page, Headings, subheadings, and file naming were revised. Please see the manuscript with track changes.

2. Thank you for stating the following in the Acknowledgments/Funding Section of your manuscript:

“This study was funded by Bill & Melinda Gates Foundation (OPP1208706). The funder had no role in study design, data collection, data analysis, data interpretation, or writing of the manuscript. The corresponding author had full access to all the data in the study and had final responsibility for the decision to submit for publication.”

We note that you have provided funding information that is not currently declared in your Funding Statement. However, funding information should not appear in the Acknowledgments section or other areas of your manuscript. We will only publish funding information present in the Funding Statement section of the online submission form.

Please remove any funding-related text from the manuscript and let us know how you would like to update your Funding Statement. Currently, your Funding Statement reads as follows:

“This study was funded by Bill & Melinda Gates Foundation (OPP1208706). The funder had no role in study design, data collection, data analysis, data interpretation, or writing of the manuscript. The corresponding author had full access to all the data in the study and had final responsibility for the decision to submit for publication.”

Please include your amended statements within your cover letter; we will change the online submission form on your behalf.

Response: Funding information removed from the manuscript text and the statement included in the cover letter as per the comment.

3. Thank you for stating the following in your Competing Interests section:

“We declare that there is no any conflict of interest that might have influenced the performance and the presentation of the work described in this manuscript.”

Please complete your Competing Interests on the online submission form to state any Competing Interests. If you have no competing interests, please state ""The authors have declared that no competing interests exist."", as detailed online in our guide for authors at <http://journals.plos.org/plosone/s/submit-now>

 This information should be included in your cover letter; we will change the online submission form on your behalf.

Response: Competing interest included in the cover letter.

4. Please include a caption for figure 2.

Response: caption included for figure 1 and 2.

5. Please upload a copy of Figure 1-3, to which you refer in your manuscript. If the figure is no longer to be included as part of the submission please removes all reference to it within the text.

Response: Our manuscript has two figures (Fig 1and 2) and two tables (Table 1 and 2) and all uploaded.

Please review your reference list to ensure that it is complete and correct. If you have cited papers that have been retracted, please include the rationale for doing so in the manuscript text, or remove these references and replace them with relevant current references. Any changes to the reference list should be mentioned in the rebuttal letter that accompanies your revised manuscript. If you need to cite a retracted article, indicate the article’s retracted status in the References list and also include a citation and full reference for the retraction notice.

Response: Reference checked for retraction and no retracted article found.

II. Reviewers' comments:

Reviewer's Responses to Questions

**Comments to the Author**

1. Is the manuscript technically sound, and do the data support the conclusions?

The manuscript must describe a technically sound piece of scientific research with data that supports the conclusions. Experiments must have been conducted rigorously, with appropriate controls, replication, and sample sizes. The conclusions must be drawn appropriately based on the data presented.

Reviewer #1: Yes

Reviewer #2: Yes

2. Has the statistical analysis been performed appropriately and rigorously?

Reviewer #1: Yes

Reviewer #2: Yes

3. Have the authors made all data underlying the findings in their manuscript fully available?

The [PLOS Data policy](http://www.plosone.org/static/policies.action#sharing) requires authors to make all data underlying the findings described in their manuscript fully available without restriction, with rare exception (please refer to the Data Availability Statement in the manuscript PDF file). The data should be provided as part of the manuscript or its supporting information, or deposited to a public repository. For example, in addition to summary statistics, the data points behind means, medians and variance measures should be available. If there are restrictions on publicly sharing data—e.g. participant privacy or use of data from a third party—those must be specified.

Reviewer #1: Yes

Reviewer #2: No

Response: We declared data availability in the online submission form. We can share data upon request

‘’Do the authors confirm that all data underlying the findings described in their manuscript are fully available without restriction? “

“Yes - all data are fully available without restriction’’

4. Is the manuscript presented in an intelligible fashion and written in standard English?

PLOS ONE does not copyedit accepted manuscripts, so the language in submitted articles must be clear, correct, and unambiguous. Any typographical or grammatical errors should be corrected at revision, so please note any specific errors here.

Reviewer #1: Yes

Reviewer #2: Yes

5. Review Comments to the Author

Please use the space provided to explain your answers to the questions above. You may also include additional comments for the author, including concerns about dual publication, research ethics, or publication ethics. (Please upload your review as an attachment if it exceeds 20,000 characters)

Reviewer #1: It was a pleasure to review the manuscript by Meaza et al. on the accuracy of the Truenat TB & RIF resistance assays in Addis Ababa, Ethiopia. Their study results contribute to the important discourse on the increasing need to promptly detect tuberculosis and drug resistance to allow the institution of appropriate management measures early. The manuscript is well written, the methods and reference standards are adequate, as are the results and discussion. The authors also briefly alluded to the limitations of the study. Below are some comments that the authors may wish to address:

1. Line 57: change ‘Truent’ to “Truenat”

Response: Thank you. Corrected as indicated in line 60 in track changes.

2. Line 58: add “s” to sample to read “samples”

Response: Corrected as indicated in line 62 in track changes.


3. Line 104: change ‘...assays is not studied...’ to “assays have not been studied” as appropriate.

Response: Corrected as indicated in line 107 in track changes.

4. Line 110: How many and how were microscopy centers selected? Also, how were the participants selected (inclusion criteria) and recruited, e.g., consecutively, or randomly?

Response: We thank the reviewer for this comment. The sites are selected based on their number of sputum smear examination, number of smear positivity rate and unavailability of GeneXpert.

The study enrolled 200 adults with clinical suspicion of pulmonary TB who were 18 years or older, willing to provide four rounds of sputum samples and willing to provide informed consent as inclusion criteria. Eligible study participants who were willing to participate in the study recruited consecutively.

Revised as indicated in line 114-119 in track changes.

5. Lines 185-188: Why were pooled samples only analyzed at the reference laboratory and not at the microscopy centers as well? Doing so would have provided extra information on the diagnostic proficiency at the microscopy centers.

Response: We thank the reviewer for this comment.

Four sputum samples were collected from each eligible study participant, two spot sputum samples in the first-day visit and pooled for Truenat, Xpert, and Culture in the reference laboratory. Two sputum samples (one morning-for culture in the reference lab and one spot for Truenat in microscopy center) in the second-day visit.

Pooling of two sputum samples could not be performed in the microscopy center where the biosafety level of the laboratory is minimal (BSL 1). This procedure was performed in the reference laboratory in Biological safety cabinet (BSL 2 or 3)

The purpose of analyzing pooled samples only in the reference lab was to see the diagnostic accuracy of Truenat from the pooled samples over direct samples. The diagnostic accuracy showed lower sensitivity for Truenat MTB and higher sensitivity for MTB Plus assay in the reference laboratory than microscopy centers as indicated in line 166-170 in track changes.

6. Lines 192-193: Can the authors consider deleting “slightly” considering it may be a stretch to characterize the difference between 50% and 86.2% as slight?

Response: Yes, well appreciated for the suggestion and deleted as indicated in line 208 in track change.

7. Lines 238-240: This conclusion may be presumptuous because data reported herein does not demonstrate the positivity levels of the ZN slides, e.g., 1+, 2+ or 3+? Including this data would have bolstered the author’s assertion. Can the authors comment on this or adjust their conclusion as appropriate?

Response: We thank the reviewer for this comment.

The study aimed to determine the sensitivity and specificity of Truenat assays in the detection of TB and RIF resistance detection in the microscopy centers. The reference standard and comparator standard are culture/DST and Xpert MTB/RIF respectively. Truenat assays were not planned to be compared to ZN smear microscopy as the sensitivity of sputum smear microscopy is less (60%, Umair M. Diagnostic Accuracy of Sputum Microscopy in Comparison With GeneXpert in Pulmonary Tuberculosis. Cureus. 2020 Nov 8;12(11):e11383. doi: 10.7759/cureus.11383). Microscopy centers were selected as the study area because they are the primary health settings where the Truenat assay planned to be evaluated and further implemented.

One of the challenges of Trueat assay in our study was high number of invalid rates. The overall non-determinate rate of Truenat assays was high (14.5%) compared to the invalid rate of Xpert MTB/RIF assay (3.7%) from direct sputum samples as indicated in table 2. This resulted in repeated testing which increases the cost and turnaround time.

Therefore we recommended that the manufacturer should improve the assays to reduce the non-determinate results.

8. If the intention of the authors is to introduce the use of Truenat tests widely in Ethiopia, did the authors consider using sentinel sites throughout the Country instead of the capital city alone?

Response: Yes we considered to implement and we appreciate for the comment. We considered 15 sentinel sites throughout the country to implement the testing and to see the operational feasibility of Truenat testing. For the purpose of evaluation study we used sites from the capital city alone which are near to the reference laboratory (for the reference and comparator standards) to transport the sample and to run the testing with the same day of sample collection.

Reviewer #2: The manuscript by Abyot Meaza et al., entitled “Diagnostic accuracy of Truenat Tuberculosis and Rifampicin- Resistance assays in Addis Ababa, Ethiopia” report the comparative analysis on sensitivity and specificity between the recently introduced Trenat-Tb assays with the previously known XpertTB assay. As per the data collected from 200 patients from Ethiopia, two of the three Truenat assay platforms (MTB Plus and MTB-RIF Dx) were found to be highly comparable to XpertTb assay in terms of specificity and sensitivity. The authors suggest the potential of Truenat assays in future point of care TB diagnostic test while accepting the limitation associated with the current small scale study. The study also recommends further standardization in reducing the relatively high rate of non-determinate or invalid results in Truenat assays.
Although a short report, the manuscript is well written and provided sufficient statistical analysis when required. Still, there are some modifications needed for the better representation of the data.
Major comments
1) The authors may consider providing a graphical description or a simple flow chart of the method as a separate figure, before describing the data.

Response: We thank for the reviewer comment.

We provided laboratory testing workflow (sample flow, method of testing in reference laboratory or microscopy center) as figure 1.

2) The methods are not well explained. Please add details on the sputum processing, volume and proportion with the liquefaction buffer etc.

Response: Thank you for the comment.

It is well explained in detail as per the comment as indicated in line 131-137.

3) The data tables are clear however, the figure may be replotted for a better representation.

Response: Thank you for the comment.

The figure replotted as indicated in Figure 2.

Minor comments
1) Line 57, 184: Correct the spelling of “Truent”

Response: corrected as indicated in line 60 and 199 in track changes.

2) Line 91: remove “highly”

Response: Removed as indicated in line 95 in track changes.

3) Line 102-104: Combine the sentences.

Response: combined as indicated in line 106-107in track changes.

6. PLOS authors have the option to publish the peer review history of their article ([what does this mean?](https://journals.plos.org/plosone/s/editorial-and-peer-review-process#loc-peer-review-history)). If published, this will include your full peer review and any attached files.


If you choose “no”, your identity will remain anonymous but your review may still be made public.


**Do you want your identity to be public for this peer review?** For information about this choice, including consent withdrawal, please see our [Privacy Policy](https://www.plos.org/privacy-policy).

Reviewer #1: **Yes:** Isdore Chola Shamputa

Reviewer #2: **Yes:** Jees Sebastian

[NOTE: If reviewer comments were submitted as an attachment file, they will be attached to this email and accessible via the submission site. Please log into your account, locate the manuscript record, and check for the action link "View Attachments". If this link does not appear, there are no attachment files.]

While revising your submission, please upload your figure files to the Preflight Analysis and Conversion Engine (PACE) digital diagnostic tool, <https://pacev2.apexcovantage.com/>. PACE helps ensure that figures meet PLOS requirements. To use PACE, you must first register as a user. Registration is free. Then, login and navigate to the UPLOAD tab, where you will find detailed instructions on how to use the tool. If you encounter any issues or have any questions when using PACE, please email PLOS at [figures@plos.org](mailto:figures@plos.org). Please note that Supporting Information files do not need this step.
